# Spontaneous signal generation by an excitable system for cell migration

**DOI:** 10.3389/fcell.2024.1373609

**Published:** 2024-02-28

**Authors:** Satomi Matsuoka, Koji Iwamoto, Da Young Shin, Masahiro Ueda

**Affiliations:** ^1^ Laboratory of Single Molecule Biology, Graduate School of Frontier Biosciences, Osaka University, Osaka, Japan; ^2^ Laboratory of Single Molecule Biology, Department of Biological Sciences, Graduate School of Science, Osaka University, Osaka, Japan; ^3^ Laboratory for Cell Signaling Dynamics, Center for Biosystems Dynamics Research (BDR), RIKEN, Osaka, Japan

**Keywords:** excitable system, spontaneous signal generation, Ras, random cell migration, chemotaxis

## Abstract

Eukaryotic cells exhibit random migration in the absence of extracellular directional cues. This random migration acts as basal motility for various migratory responses such as chemotaxis. The self-organization of random motility requires the internal signals that determine the anterior side of the migrating cell be generated spontaneously from the intrinsic activities of intracellular signaling networks. Recent studies have identified an excitable system as the mechanism of the spontaneous signal generation. Here, we discuss how the excitable system of Ras, a small G protein, regulates signaling networks in *Dictyostelium discoideum* as a model organism. The excitability produces a domain where an active form of Ras is enriched on the cell membrane without extracellular directional cues, such that Ras serves as the anterior signal. The typical spatiotemporal characteristics are mathematically explained by reaction-diffusion models. These models further enable a quantitative analysis of the dynamics that depends on the internal cellular states and surrounding environments. Downstream of the Ras excitable system, a phosphoinositide metabolic network composed of PI3K, PTEN, PI(3,4,5)P_3_ and PI(4,5)P_2_ exhibits bistability to discretize the anterior and posterior regions of the cell membrane. Upstream, a local excitation and global inhibition local excitation global inhibition network, which works for gradient sensing in the presence of chemoattractant gradients, spatiotemporally biases the excitability of Ras for chemotaxis. In parallel with the Ras excitable system, the cGMP signaling pathway constitutes another excitable system of its own periodicity to ensure flexible migratory dynamics. In addition to these intracellular signaling networks, an intercellular signaling network activated by secreted cAMP is coupled with the Ras excitable system for collective cell migration. Finally, we discuss how the excitable system of Ras operates as a platform of information integration by receiving multiple intrinsic and extrinsic signals to ensure spontaneous cellular activity and robust responses in eukaryotic cell migration under natural complex environments.

## 1 Introduction

Migration is one of the fundamental means living cells use to escape from unfavorable environments and seek better ones. Vast and complicated molecular networks of signal transduction have developed during evolution, enabling cells to respond adaptively to various environmental changes. One such survival strategy is cellular taxis, in which cells adapt to move positively or negatively in response to guidance cues in the environment, including chemicals, electric fields, temperature, light and so on. Of these cell responses, chemotaxis is the best understood in terms of its molecular mechanisms. One of the most sophisticated responses seen in mammalian immune systems is the chemotaxis of neutrophils promptly chasing foreign materials such as bacteria, but chemotaxis is observed ubiquitously among a wide range of eukaryotes, and the basic mechanism is conserved evolutionally (Graziano and Weiner, 2014; SenGupta et al., 2021). The molecular network of chemotactic signal transduction has been elucidated by exploiting the social amoeba *Dictyostelium discoideum* as a model organism (Devreotes et al., 2016; Liu et al., 2016; Haastert et al., 2021; Nichols et al., 2015; Stuelten et al., 2018; Xu and Jin, 2022). In this organism, the molecular network for chemotaxis also can work to regulate motile behaviors in response to electric fields and sheer stress and even in the absence of any guidance cues (Zhao et al., 2006; Sato et al., 2009; Artemenko et al., 2016). That is, the chemotactic signaling system can regulate directional cell migration as well as spontaneous random cell migration depending on the surrounding environment and cellular internal state. Under natural complex environments, living cells often encounter environmental stimulations simultaneously to which they respond for their survival. Living cells have molecular mechanisms for integrating various information from the environment and their own state to determine the direction of movement, but these mechanisms are poorly understood. Motile cells have an anterior-posterior polarity along their length, ensuring robust directed migration with two opposing signals simultaneously at both their ends: one that regulates anterior pseudopods and another that regulates posterior tails. Motile cells determine their anterior-posterior polarity and migrate using spatially distinct signaling generated by the intracellular signaling system. To understand the mechanisms by which motile cells make decisions for their migration direction via information integration, it is necessary to clarify how their anterior-posterior polarity is self-organized spontaneously or in an environment-dependent manner.

The anterior-posterior polarity is generated spatially in an all-or-none manner even if the extracellular information is uncertain. Many types of eukaryotic cells can exhibit migration even in the absence of extracellular information, a phenomenon known as spontaneous migration. There is no preferred direction of migration under a homogeneous environment, and at the single-cell level, the migration involves spontaneous symmetry breaking, such that the cell moves in one direction without extracellular cues. To achieve the polarity independently of ligand binding and thus receptor activation, the intracellular signaling system itself can self-organize a domain where anteriorly-working signaling molecules are enriched on the cell membrane (Arai et al., 2010; Postma et al., 2004; Sasaki et al., 2007). Recent studies have revealed that the mechanistic basis for the self-organization is provided by an excitable system, which prescribes the spatiotemporal characteristics of the domain intrinsically (Nishikawa et al., 2014). Upon the spontaneous migration and chemotaxis of amoeboid cells, a single dominant pseudopod with a dense F-actin meshwork protrudes and produces a force to move forward. In the absence of cues, each single pseudopod is transient, and the leading edge is taken over by a newly formed lateral pseudopod successively. In the presence of guidance cues, the dominant pseudopod is stable and the lateral pseudopod is suppressed (Swanson and Taylor, 1982; Varnum-Finney et al., 1987). The morphology of the pseudopod is almost constant irrespective of the extracellular cues. As suggested by these observations, chemotaxis is achieved by biasing the spontaneously generated signals and thus the pseudopod directionally, and the role of the chemoattractant gradient is to reinforce the direction of the anterior-posterior polarity. Thus, the mechanisms of spontaneous signal generation and directional bias via the integration of environmental information by the excitable system determine the motile behavior of cells.

Excitable systems have been given much attention as the core of intracellular signaling. This review discusses the self-organization of the anterior-posterior polarity of migrating cells by an excitable system with emphasis on the molecular network of the excitable system and spatiotemporal regulations for efficient cell migration. Also introduced are studies concerning biological functions underpinned by excitability, how cell-to-cell variability in cell migration arises from a common excitable system, how parallel signaling pathways individually constituting an excitable system provide flexible responses in concert, and how multiple excitable systems are coupled in collective cell migration to produce consistent migratory behaviors between single cells and cell mass.

## 2 Emergence of asymmetric signals by excitability

The asymmetric localization of signaling molecules along cellular anterior-posterior polarity was first discovered in the pleckstrin homology domain (PHD)-containing protein CRAC (cytosolic regulator of adenylyl cyclase) (Parent et al., 1998; Weiner et al., 2002). The PI(3,4,5)P_3_-binding activity of PHD led to the identification of the metabolizing enzymes PI3K and PTEN, which are now widely accepted as representative components of the signaling pathways essential for cell motility including chemotaxis and spontaneous migration (Funamoto et al., 2002; Iijima and Devreotes, 2002). PI3K was also shown to be essential for polarity maintenance and robust directed motility in mammalian cells (Wang et al., 2002). These almost simultaneous reports by independent research groups demonstrate that PI3K and its product PI(3,4,5)P_3_ are generally conserved molecules that determine the moving direction in eukaryotic systems.

**FIGURE 1:**
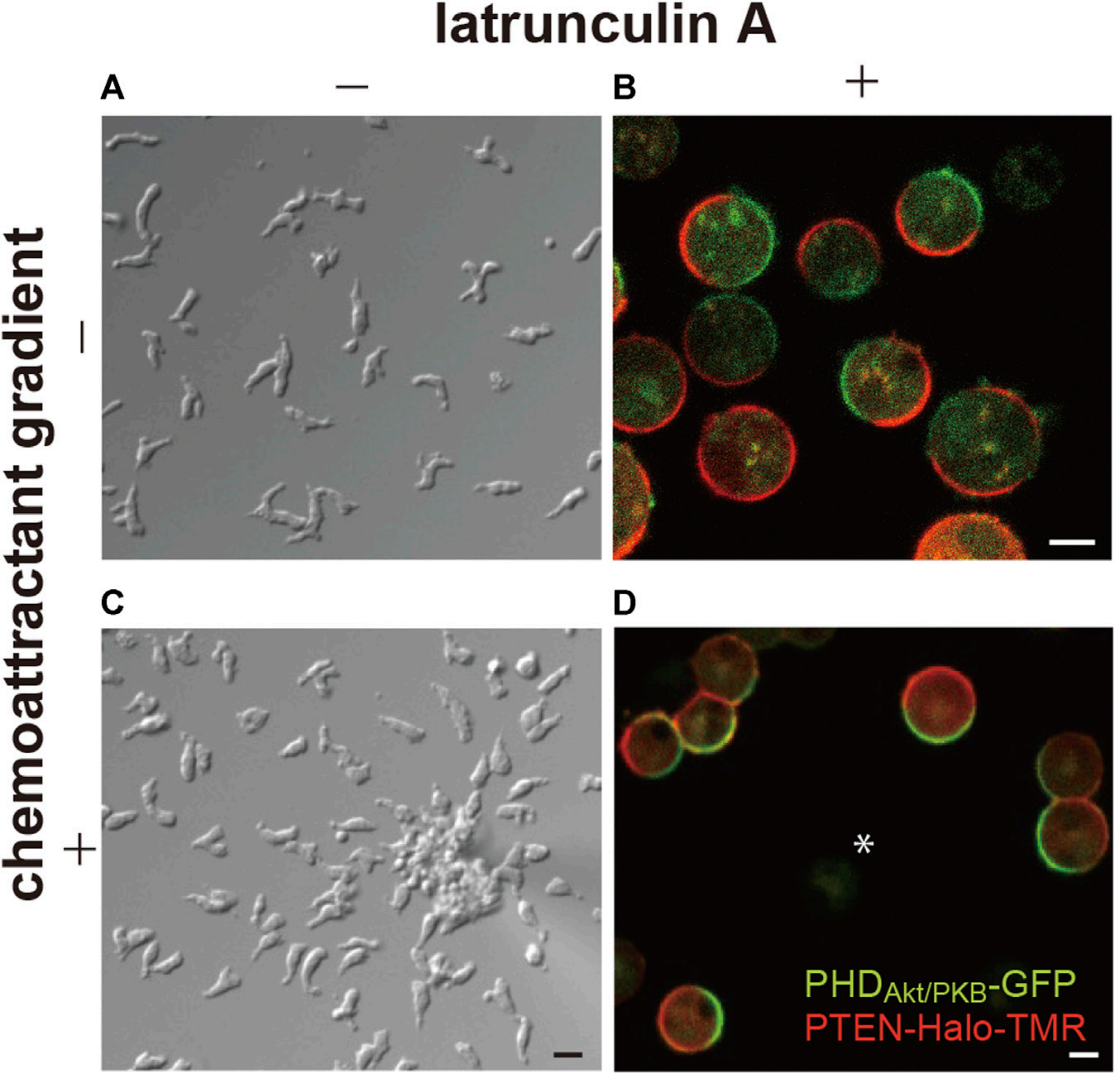
Spontaneous generation of asymmetric signals for anterior-posterior polarity and cell motility. **(A)** A representative image of *Dictyostelium discoideum* cells undergoing spontaneous migration. **(B)** A representative image of the cells treated with latrunculin **(A, C)** A representative image of the cells undergoing chemotaxis toward cAMP. **(D)** A representative image of the cells treated with latrunculin A in the presence of a cAMP gradient. Scale bars, 10 μm **(A, C)** and 5 μm **(B, D)**.

Additionally, these reports showed patches, or domains, of PHD-containing proteins localize on the membrane at the side facing the higher concentration of the chemoattractant (Figure 1). It is widely known that the domains are generated not only in response to chemoattractant gradients but also in a spontaneous manner at the leading edge of the migrating cells (Sasaki et al., 2007). Furthermore, the self-organization of the PI(3,4,5)P_3_-enriched domains is independent of the actin cytoskeleton. In latrunculin A-treated cells, the domains are generated transiently at random times and positions on the cell membrane, although they become continuous in the presence of a chemoattractant (Postma et al., 2004; Arai et al., 2010; Nishikawa et al., 2014). Stochastically the generated domains share stereotypical temporal and spatial features, suggesting that each domain arises from an excitable system.

Excitable systems in biology were originally found in action potentials (Hodgkin and Huxley, 1952), which exhibit a stereotypical pattern of an increase and decrease in membrane potential that propagates from the soma to axon terminal in neuronal cells. Since then, they have been found in a variety of living systems, from bacteria to mammals. Min proteins exhibit oscillations from pole to pole in *Escherichia coli* to properly localize the cell division machinery (Merino-Salomón et al., 2021). Cortical actin patterns are commonly seen in eukaryotic cells through the coupling of actomyosin dynamics and membrane deformation (Yang and Wu, 2018). During embryogenesis, mitotic waves are seen in the embryos of *Drosophila melanogaster* and *Xenopus laevis*, where the mitosis of neighboring cells is synchronized and occurs in a wave-like pattern due to Cdk1 activity (Talia and Vergassola, 2022).

Excitable dynamics in organisms depend on biochemical reactions or mechanochemical processes (Wu and Liu, 2021). For simplicity, two feedback regulations are generally assumed to explain the operation principle of an excitable system (Goldbeter, 2010). A stereotypical response is generated through both positive and negative feedback to regulate the level of a certain signal such as the membrane potential. At the onset of excitation, the signal level crosses a threshold to trigger positive feedback, which amplifies the signal itself. The threshold crossing, however, also initiates a delayed negative feedback, which diminishes the signal so that the signal level autonomously returns to its initial level. In general, an excitable system exhibits three characteristic features. First, there is a threshold in the excitable system so that the system behaves in an all-or-none manner; the system only exhibits an excitation when it reaches the suprathreshold state. Threshold crossing can be caused either by extrinsic stimulations or by intrinsic fluctuations contained in the system itself. Second, an excitation undergoes stereotypical dynamics. The amplitude of the response is constant irrespective of prolonged or pulsatile stimulations, but the spatiotemporal properties of the excitation are modulated by the magnitude of the stimulation. For example, greater stimulations increase the frequency of action potentials and the size of PI(3,4,5)P_3_-enriched domains (Nishikawa et al., 2014; Tanabe et al., 2018). Spontaneous excitation caused by intrinsic fluctuations has similar dynamics. Third, an excitation is followed by a refractory period. The excitation cannot be triggered during this period even in the presence of sufficiently large stimulations. After the refractory period, the system autonomously becomes capable of exhibiting the next excitation. The generation of the PI(3,4,5)P_3_-enriched domain shares these features of an excitable system (Nishikawa et al., 2014). Additionally, the chemotactic signaling system of *D. discoideum* cells exhibits all of the typical spatiotemporal dynamics seen in excitable systems.

**FIGURE 2:**
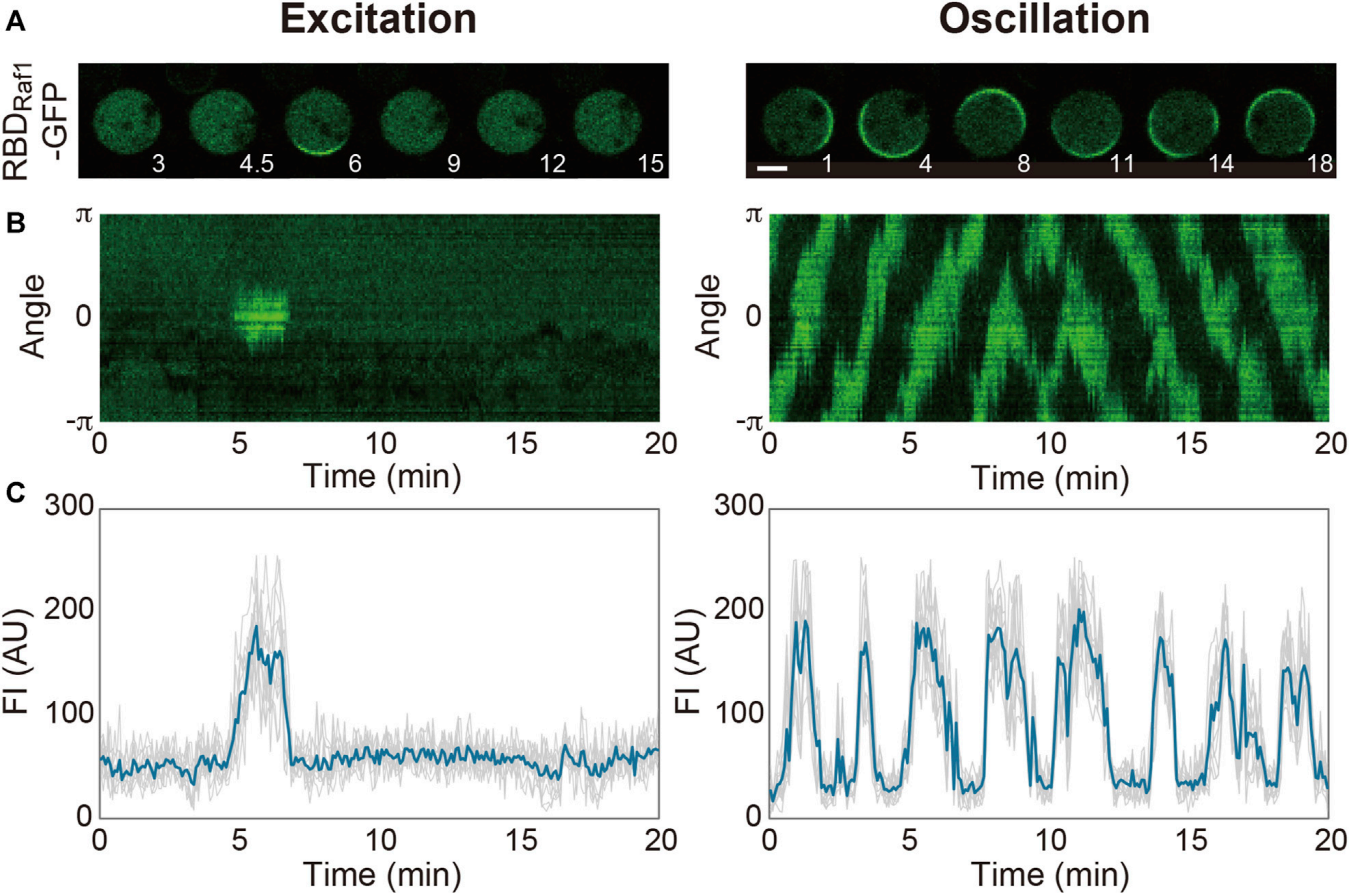
Spontaneous dynamics of the Ras excitable system. **(A)** Representative time-lapse images of *Dictyostelium discoideum* cells treated with latrunculin A in the absence (left) and presence (right) of caffeine. **(B)** Representative kymographs acquired along the periphery of the cell images in **(A)**. **(C)** Time series of the fluorescence intensities of RBD_Raf1_-GFP. Scale bar, 5 μm. Time, min.

Spontaneous dynamics in the absence of external stimulations is largely dependent on the intrinsic state of the system and prescribed by physicochemical parameters such as the concentrations of the system’s components, cell volume and membrane area. Even in a genetically identical ensemble of *D. discoideum* cells grown in the same medium, there are cell-to-cell variations in these parameters and thus the dynamics (Figure 2). The transient domains appear and disappear at random locations on the cell membrane at high frequencies in some cells, while they hardly appear in other cells. Under our experimental conditions, 37% of *Dictyostelium* cells frequently show domain generation (Nishikawa et al., 2014): the frequency of domain generation is an indicator of the excitability of the system in an individual cell. When the system is set close to the threshold so that it crosses the threshold easily, frequent excitation occurs. The distance to the threshold is shortened upon uniform stimulation with a chemoattractant (in this case, cAMP), which increases the above percentage to 65%. Under conditions that enhance excitability, the excitation occurs soon after the refractory period is over, leading to regularly repeated excitations, i.e., an oscillation. Experimentally, the transition of the dynamics from excitation to oscillation can be induced by exogenously adding caffeine. In the presence of 4 mM caffeine, a PI(3,4,5)P_3_-enriched domain propagates continuously as a traveling wave on the cell membrane. A traveling wave is observed in most (around 80%) cells with caffeine, but it is seldom observed without caffeine (Arai et al., 2010; Shibata et al., 2012). It is also reported that the membrane translocation of inositol polyphosphate 5-phosphatase (Inp54p), a yeast PI(4,5)P_2_-specific phosphatase, via chemically induced dimerization shifts the mode from excitation to oscillation (Miao et al., 2017). Because a statistical analysis of the spatiotemporal dynamics is easier to perform with traveling waves, caffeine is often used to enhance excitability in experiments.

## 3 Molecular network of the excitable system

### 3.1 Excitability of Ras

**FIGURE 3:**
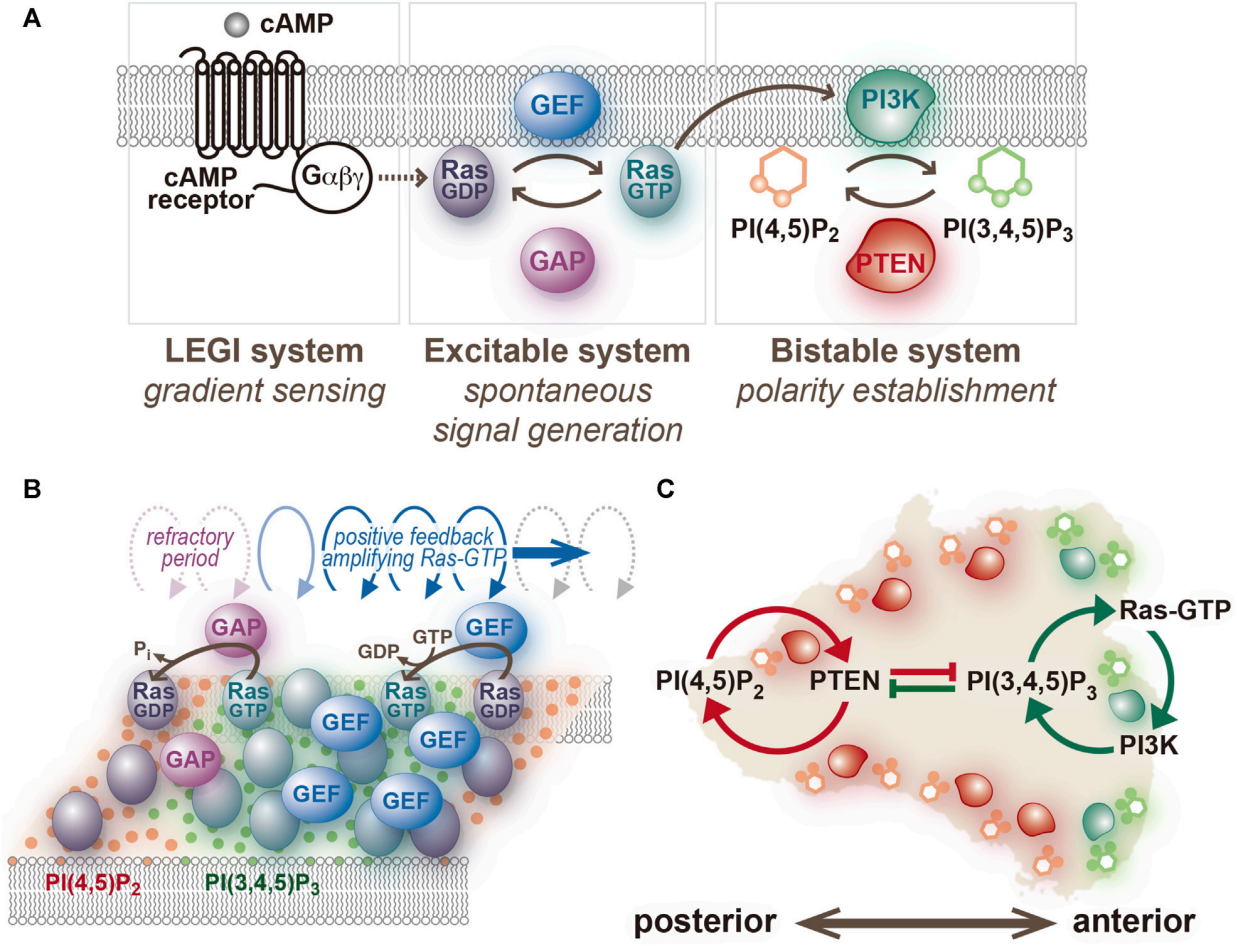
An excitable system of Ras and a bistable system of PI(3,4,5)P_3_/PTEN. **(A)** The network structure assumed in the present model. **(B)** A schematic representation of traveling wave propagation through cooperative Ras activation and inactivation. **(C)** A schematic representation of anterior-posterior polarity formation through mutual inhibition between PI(3,4,5)P_3_- and PTEN-dependent positive feedback loops.

Multiple lines of evidence suggest that a central component of the Ras excitable system, a small GTPase, and not PI(3,4,5)P_3_ (Figure 3) (Fukushima et al., 2019; Shin et al., 2023). Using a GFP-tagged Ras-binding domain of c-Raf1 (RBD_Raf1_), which binds an active form of Ras (Ras-GTP) (Kae et al., 2004; Sasaki et al., 2004), traveling waves propagating on the cell membrane were visualized in *D. discoideum* cells treated with latrunculin A and caffeine. The activities of four signaling pathways that work in parallel for cell motility: the RasG/D-PI3K, RasC-TorC2, Rap1/cGMP and PLA2 pathways [Devreotes et al., 2016; Veltman et al., 2008], were all found dispensable for the generation of the Ras traveling wave (Fukushima et al., 2019). Therefore, the Ras excitable system is capable of spontaneous symmetry breaking without any downstream signaling activity, chemoattractant binding to the receptors, or remodeling of the actin cytoskeleton. The active forms of RasG, RasD and RasB, but not RasC, are detectable using the binding specificity of RBD_Raf1_ assessed biochemically or with the yeast two hybrid system (Sasaki et al., 2004; Kae et al., 2004). Among these molecules, RasG is the most potent upstream regulator of PI3K; the order of descending interaction strength with PI3K is RasG, RasD, and RasB, which is predominantly localized in the nucleus (Funamoto et al., 2002; Sutherland et al., 2001).

In *D. discoideum*, Ras is the most upstream component whose activity exhibits asymmetry and regulates PI3K on the cell membrane [Fukushima et al., 2019; Devreotes et al., 2016]. In other eukaryotic systems, such as human HT-1080 fibrosarcoma cells and mouse embryonic fibroblast as well as yeasts, Rac, Cdc42 and Rho are the primary components for cellular polarity formation; their activities distribute asymmetrically and regulate F-actin dynamics directly (Pertz et al., 2006; Yamao et al., 2015; Heasman and Ridley, 2008; Thompson, 2013). Symmetry breaking by other small GTPases likely depends on actin cytoskeletal activity, including force generation and membrane tension (Paluch et al., 2006; Houk et al., 2012). However, whether asymmetry in the activities of these molecules arises in the presence of actin polymerization inhibitors requires further investigation.

The traveling wave is dependent on the perpetual activation and inactivation of Ras, which take place in a spatiotemporally cooperative manner. Notably, the wave does not reflect individual Ras-GTP molecules changing their locations in the same direction on the cell membrane. Instead, Ras activation is promoted at the front of the traveling wave and its inactivation is prominent at the back (Figure 3). Therefore, the traveling wave requires activities of both guanine nucleotide exchange factor (GEF) and GTPase-activating protein (GAP). Previous studies have demonstrated that RasGEFR and NF1 contribute to the activation and inactivation of RasG, respectively (Kae et al., 2004; Zhang et al., 2008). However, investigations so far have been limited to their roles in regulating Ras activity in response to chemoattractant stimulation, and their roles in spontaneous cell motility remain to be elucidated. Moreover, there are 25 and 14 subtypes of RasGEF and RasGAP encoded in the *D. discoideum* genome, respectively (Wilkins et al., 2005), but their contributions to the Ras excitable system are required to be assessed systematically.

Ras is an activator of class I PI3K and triggers the traveling wave of signaling molecules of the PI3K pathway (Pacold et al., 2000). Under confocal laser scanning microscopy (CLSM), fluorescently labeled PI3K2, a subtype of class I PI3K that makes the largest contribution in PI(3,4,5)P_3_ production and chemotactic signaling among 6 PI3K subtypes in *D. discoideum*, is hardly detectable on the cell membrane (Funamoto et al., 2002; Takeda et al., 2007). However, total internal reflection fluorescence microscopy (TIRFM) overcomes this detection problem with an improved signal-to-noise ratio achieved by limited excitation of cell membranes near the glass surface (Axelrod, 1984; Sako et al., 2000; Ueda et al., 2001). Under TIRFM, two-dimensional propagation of the traveling wave is observed on the membrane plane, where RBD_Raf1_-RFP and PI3K2-GFP exhibit co-localization (Fukushima et al., 2019). It is most likely that Ras-GTP recruits both these proteins via RBDs. Mammalian class I PI3K works as a dimer composed of the catalytic subunit p110α, which contains RBD, C2 domain and PI3K catalytic domain, and of the regulatory subunit, p85α (Vanhaesebroeck and Waterfield, 1999; Cantley, 2002; Huang et al., 2007). *Dictyostelium discoideum* PI3K2 works as a monomer that shares a conserved domain structure with p110α (Funamoto et al., 2002). Instead of p85α, PI3K2 contains an N-terminal domain for membrane targeting; this domain shows no obvious sequence similarity to known proteins. Even though the secondary structures are different, the lysines at residues 858 in PI3K2 and 227 in human p110α are completely conserved; lysine is a key residue in human p110α for the formation of salt bridges with aspartic acids at residues 33 and 38 in KRas4B [Zhang et al., 2019]. The preceding biochemical studies revealed that lysines 857 and 858 in PI3K2 RBD are essential for the interaction between RasG-GTP and PI3K2 (Funamoto et al., 2002). Importantly, the amino acid substitution to glutamate (K857E/K858E) in PI3K2 eliminates PI3K2 and PI(3,4,5)P_3_ traveling waves. These results suggest that the excitability of PI3K is subordinate to that of Ras.

**FIGURE 4:**
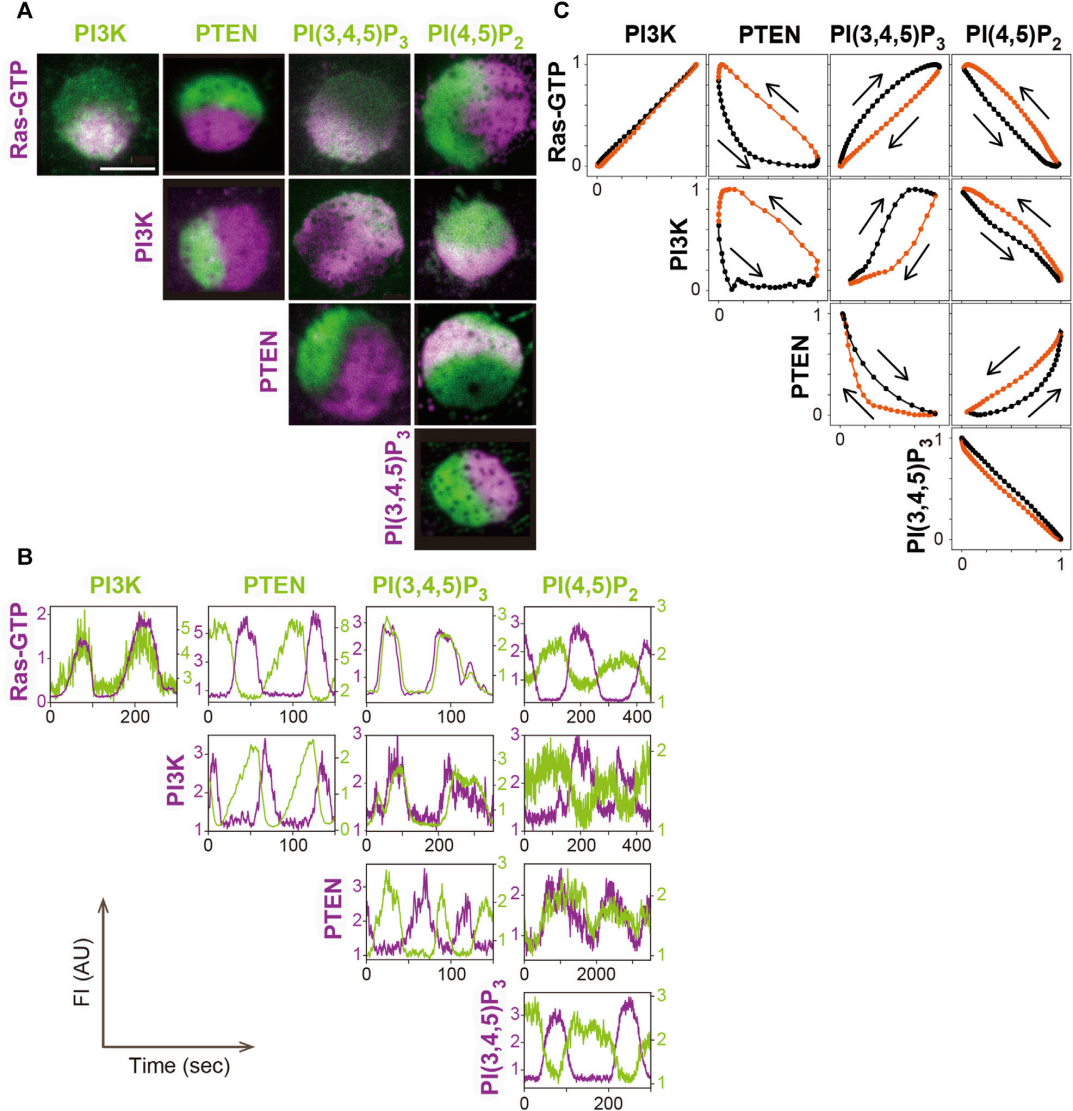
A systematic analysis of the excitable dynamics of traveling waves. **(A)** Simultaneous imaging of signaling molecules in the excitable-bistable systems. **(B)** Time series of the traveling wave propagation. **(C)** The trajectory of one cycle of the traveling wave shows changes in the fluorescence intensity. Scale bar, 5 μm. Reproduced with permission from Fukushima et al., 2019.

The amounts of PI(3,4,5)P_3_, PI(4,5)P_2_ and PTEN change at the same time as the amount of PI3K in the traveling wave (Figure 4). Through fluorescent labeling of two arbitrary molecules with different colored dyes, simultaneous live-cell imaging revealed how the changes are interrelated (Arai et al., 2010; Fukushima et al., 2019). Fluorophores such as GFP and tetramethylrhodamine (TMR), which is tagged to the protein of interest via HaloTag protein, have been proven to be useful for such imaging (Arai et al., 2010). PI(3,4,5)P_3_, detected with the PHD of PKB/Akt (PHD_PKB/Akt_) (Asano et al., 2004), increases and decreases with a slight delay to the increase and decrease of PI3K, respectively. PI(4,5)P_2_, detected with the Nlj6-like nodulin domain of *Arabidopsis* AtSfh1 Sec14-nodulin protein or PHD of PLCδ (PHD_PLCδ_) (Harlan et al., 1994; Ghosh et al., 2015; Miao et al., 2017), changes coincidently with PI(3,4,5)P_3_ but in the opposite direction. Although PI(3,4,5)P_3_ is contained at a far lower level than PI(4,5)P_2_ on the cell membrane (Clark et al., 2014), the PI(3,4,5)P_3_ probe is sensitive enough to detect the slight increase. The relative changes of the PI(3,4,5)P_3_ and PI(4,5)P_2_ probes are complementary to each other during the traveling wave propagation. This relationship suggests that metabolic pathways of phosphoinositide other than 3′ phosphorylation and dephosphorylation, such as 5’ dephosphorylation of PI(3,4,5)P_3_ by Src homology 2 (SH2) domain, which contains inositol polyphosphate 5-phosphatase (SHIP), make insignificant contributions to the traveling wave propagation (Damen et al., 1996; Ware et al., 1996). The amount of PTEN decreases with a delay in response to the increase and decrease of PI(3,4,5)P_3_ and PI(4,5)P_2_, respectively, due to negative feedback by the substrate and positive feedback by the product, as discussed below.

Statistical analysis of the relationship between PI(3,4,5)P_3_ and PTEN in the traveling waves revealed the stereotypical trajectory of a crescent shape plotted in the PI(3,4,5)P_3_-PTEN plane (Arai et al., 2010; Shibata et al., 2012). This trajectory shows the characteristics of a relaxation oscillation that contains two relatively stable states: a high PI(3,4,5)P_3_ and low PTEN state and a high PTEN and low PI(3,4,5)P_3_ state, as described below.

### 3.2 Bistability of PtdIns, PI3K and PTEN

The phosphoinositide metabolic system is responsible for separating anterior and posterior signals but is dispensable for the spontaneous symmetry breaking in Ras dynamics (Matsuoka and Ueda, 2018). Once a spontaneous excitation generates a Ras-GTP-enriched domain locally on the cell membrane, PI(3,4,5)P_3_ is produced by PI3K, and PTEN disappears simultaneously at the site (Arai et al., 2010; Fukushima et al., 2019). The PI(3,4,5)P_3_-enriched domain has a clearly recognizable border despite the lateral diffusion of phospholipid molecules being relatively fast. PTEN is enriched outside the domain to make the border clear, where PI(3,4,5)P_3_ and PTEN levels exhibit steep interchanges (Matsuoka and Ueda, 2018). The confined signal of PI(3,4,5)P_3_ induces actin polymerization for regular pseudopod extension. When the pseudopod is retracted, the anterior signaling molecules disappear from the cell membrane, and PTEN is simultaneously recruited to occupy the whole cell membrane. These processes dominating cellular spontaneous migration occur independently of chemoattractant stimulation, indicating that the temporally and spatially interchanging membrane localization of PI(3,4,5)P_3_ and PTEN is regulated autonomously. Such switch-like behavior is characteristic of a bistable system. In general, a bistable system is composed of two positive feedback loops that are mutually inhibitory (Figure 3). In migrating cells, when the PTEN level increases, the PI(4,5)P_2_ level increases, which leads to further membrane localization of PTEN at the site (Matsuoka and Ueda, 2018). On the other hand, when the PI(3,4,5)P_3_ level increases, the PTEN level decreases, which decelerates PI(3,4,5)P_3_ dephosphorylation and further increases PI(3,4,5)P_3_ in the presence of PI3K activity. Thus, two positive feedback loops are assigned to the anterior and posterior signaling molecules, enabling stringent signal separation in time and space and dynamic signal transmission from upstream Ras-GTP to downstream actin polymerization. Mutual inhibition is a universal mechanism of polarity establishment, as seen in partitioning defective (PAR) proteins in *Caenorhabditis elegans* embryos (Hoege and Hyman, 2013).

In the phosphoinositide metabolic system, PI(3,4,5)P_3_ and PTEN exhibit mutual inhibition (Matsuoka and Ueda, 2018). That is, PTEN and PI(3,4,5)P_3_ exclude each other from the cell membrane. This result is expected since PI(3,4,5)P_3_ is reduced due to the dephosphorylation by PTEN. On the other hand, it is somewhat counterintuitive that PTEN is excluded from the membrane due to PI(3,4,5)P_3_, the substrate, but this effect was demonstrated by the direct manipulation of PI(3,4,5)P_3_ levels in living *D. discoideum* cells (Matsuoka and Ueda, 2018). A reversible increase and decrease in the PI(3,4,5)P_3_ level was achieved by the treatment and wash-out of a PI3K inhibitor, LY294002, in cells over-expressing PI3K2 tagged with a myristoylation signal peptide from chicken c-Src, in which the PI(3,4,5)P_3_ level is permanently increased (Sigal et al., 1994; Meili et al., 1999; Funamoto et al., 2002). In response to the PI(3,4,5)P_3_ decrease and increase, PTEN shows translocation from the cytosol to the membrane and the membrane to the cytosol, respectively (Matsuoka and Ueda, 2018). PTEN exhibits faster membrane dissociation after PI(3,4,5)P_3_ dephosphorylation than before, as revealed by directly measuring the rate constant for membrane dissociation using single-molecule imaging techniques (Matsuoka and Ueda, 2018). In addition, PTEN exhibits slower membrane association toward the PI(3,4,5)P_3_-enriched membrane (Matsuoka and Ueda, 2018). These differences in the kinetics lead to the exclusion of PTEN from the PI(3,4,5)P_3_-enriched membrane. Quantification of the lateral diffusion coefficient suggests that a certain fraction of PTEN molecules shows slow diffusion (Matsuoka and Ueda, 2018), in which the diffusion coefficient is equivalent to that of membrane-integrated proteins. This stable, slow-diffusion state of PTEN has a longer lifetime in membrane binding and is suppressed by PI(3,4,5)P_3_. The molecular identity of the membrane-binding partner of PTEN is unknown.

The positive feedback loop that amplifies PTEN involves an auto-catalytic reaction of PTEN via the product, PI(4,5)P_2_. PI(4,5)P_2_ potentiates the membrane recruitment, stable membrane binding, and catalytic activity of PTEN (Campbell et al., 2003; Walker et al., 2004; Redfern et al., 2008; Worby and Dixon, 2014). The interaction between PTEN and PI(4,5)P_2_ is driven electrostatically via the cationic surface of PTEN. This surface is composed of basic amino acid residues in a PI(4,5)P_2_-binding motif and C2 domain and contributes to specific and nonspecific electrostatic interactions with PI(4,5)P_2_ as well as other acidic phospholipids such as phosphatidylserine (PS) and phosphatidic acid (PA) (Lee et al., 1999; Das et al., 2003; Vazquez et al., 2006; Yasui et al., 2014). Molecular dynamics simulations predicted that these surface properties of PTEN are advantageous for adopting an optimal orientation of the catalytic pocket against the substrate, PI(3,4,5)P_3_, embedded in the membrane plane (Shenoy et al., 2012). These residues are conserved among various PTEN species from mammalian to social amoebae orthologues. *Dictyostelium discoideum* PTEN with substitutions of the conserved K11/K13/R14/R15/R47 into neutral amino acids fails to confine the PI(3,4,5)P_3_-enriched domain [Yoshioka et al., 2020]. Furthermore, direct measurements of the membrane-binding lifetime and lateral diffusion of PTEN by single-molecule imaging revealed that these residues are necessary for stabilizing PTEN membrane binding in a PI(4,5)P_2_ concentration-dependent manner [Yoshioka et al., 2020]. PTEN membrane binding via these electrostatic interactions promotes PI(4,5)P_2_ production on the cell membrane where PI(4,5)P_2_ is already enriched, making positive feedback an essential driving force for the establishment of the bistability.

The positive feedback loop that amplifies PI(3,4,5)P_3_ is assumed to involve an auto-catalytic reaction of PI3K via the product, PI(3,4,5)P_3_, and the upstream regulator, Ras (Heo et al., 2006; Sasaki et al., 2007). Upon the activation of Ras, PI3K is recruited to the cell membrane through RBD and produces PI(3,4,5)P_3_. It is proposed that PI(3,4,5)P_3_ in turn exerts positive feedback upon Ras-GTP to stabilize the active form. This idea is supported by the observation that the Ras-GTP-enriched domain exhibits a larger size and longer lifetime in the presence of full PI3K activity compared to conditions where PI3K is inhibited with moderate concentrations of LY294002 (Fukushima et al., 2019). The molecular basis for the positive feedback on Ras by PI(3,4,5)P_3_ has not been solved so far.

### 3.3 Modeling with reaction-diffusion equations

The dynamics of an excitable system is explained mathematically with a reaction-diffusion model. The first study of an excitable system concerned action potentials in giant axons of squids (Hodgkin and Huxley, 1952). The reaction-diffusion model, or Hodgkin-Huxley model, includes four ordinary differential equations describing the current flowing through the membrane, open probability of the sodium channel, open probability of the potassium channel, and inactivation probability of the sodium channel. How these variables change during an excitation has been described in detail in other literature (Hodgkin and Huxley, 1952; Clay, 2005). The excitable behaviors are explained well by contracted models with two variables, as represented by the Fitz-Hugh-Nagumo model (Fitzhugh, 1961; Nagumo et al., 1962). The mechanism of an excitation is essentially based on two feedback regulations, one is positive feedback that exerts fast kinetics and the other is negative feedback that exerts autonomously with a delay.

A reaction-diffusion model for the excitable system in cell migration was first constructed after the traveling waves of PI(3,4,5)P_3_ and PTEN were observed (Arai et al., 2010; Shibata et al., 2012). How the local concentrations of PI(3,4,5)P_3_, PI(4,5)P_2_ and PTEN on the cell membrane change by reactions and diffusion during an instant time interval are mathematically described with partial differential equations. The reactions include the enzymatic reactions of PI3K phosphorylating PI(4,5)P_2_ and PTEN dephosphorylating PI(3,4,5)P_3_ and the membrane association and dissociation reactions of PTEN. By incorporating both the positive and negative regulation of PTEN membrane localization by PI(4,5)P_2_ and PI(3,4,5)P_3_, the concentration of PTEN becomes dependent on time and space. These reactions are sufficient for generating two relatively stable states in the traveling wave: the PI(3,4,5)P_3_-enriched state with high PI(3,4,5)P_3_ and low PTEN levels, and the PTEN-enriched state with low PI(3,4,5)P_3_ and high PTEN levels. The model does not necessarily assume the asymmetric membrane localization of PI3K to reconstitute the bistability. By taking into account that PI3K activity is enhanced by a positive feedback loop mediated by PI(3,4,5)P_3_ and Ras-GTP, the two relatively stable states can be further stabilized.

The above reactions are sufficient for bistability but insufficient for excitability. We previously proposed a model to explain the excitation dynamics based on PI(4,5)P_2_, PI(3,4,5)P_3_, PI3K, PTEN and other enzymes (Arai et al., 2010; Shibata et al., 2012). This model can explain the travelling wave generation of PI(3,4,5)P_3_ and PTEN. However, simultaneous visualization of PI(3,4,5)P_3_ and PI(4,5)P_2_ revealed that the contribution of the reactions assumed in this model is negligible to the traveling wave generation (Fukushima et al., 2019), suggesting that the model is unlikely. A later model assumes that the excitability is independent of the PI(3,4,5)P_3_ and PI(4,5)P_2_ dynamics based on the identification of Ras being the core of the excitable system (Fukushima et al., 2019). The same scheme of fast positive and delayed negative feedback loops was adopted to explain the autonomous increase and decrease of the Ras-GTP level through RasGEF and RasGAP activities. This latest model describes spatiotemporal changes of the local concentrations of Ras-GTP, Ras-GDP and RasGAP by assuming that the local concentration of RasGAP is dependent on time and space.

Numerical simulations are a powerful tool to investigate the mechanism of how excitable systems work by predicting the dynamics under any given condition of the mathematical model and parameter values. The simplest simulation utilizes a one-dimensional system assuming only one cycle of the cell periphery is observed under CLSM (Arai et al., 2010; Xiong et al., 2010; Shibata et al., 2012; Huang et al., 2013). The essential behaviors of the system are recaptured by the simulation. Especially, the simulation reconstitutes how different dynamics, such as excitation and oscillation, arise from the same molecular network. One of the key parameters in the latest model is the basal activity of RasGEF, *V*_*GEF*_ (Fukushima et al., 2019). The excitation is observed with moderate values of *V*_*GEF*_, where Ras-GTP-enriched domains transiently appear (“transient domain”). On the other hand, the excitation is suppressed with *V*_*GEF*_ below the lower limit, where the domains hardly appear (“no domain”). Finally, the oscillation takes place with *V*_*GEF*_ above the upper limit, where the traveling wave is observed (“traveling wave”). This property is because the larger *V*_*GEF*_ causes a higher mean and larger variance of the Ras-GTP level at the resting state, leading to a higher frequency of threshold crossing. In addition, the simulation clarifies the contributions of network motifs to the dynamics of the whole system. Ras-GTP is subjected to two positive feedback loops: one is through RasGEF, and the other is through PI3K and PI(3,4,5)P_3_ (Fukushima et al., 2019). Experimental evidence supports that only RasGEF is essential for the excitation (Fukushima et al., 2019). Consistently, the three dynamics (no domain, transient domain and traveling wave) are simulated depending on *V*_*GEF*_ values even if the positive feedback via PI3K is absent, where the parameter denoting PI3K activity, *V*_*PI3K*_, equals 0. On the other hand, the transient domain and traveling wave are not simulated with any *V*_*PI3K*_ values if the positive feedback via RasGEF is absent, because *V*_*GEF*_ is below the lower limit described above.

A quantitative analysis of the mathematical model also suggests a role of PTEN as a global coupling regulator of wave number (the number of domains simultaneously generated in the cell), which is usually 1 in *D. discoideum* cells. Experiments have demonstrated that the membrane localization of PTEN is regulated via shuttling between the cell membrane and cytoplasm at the time scale of sub-seconds for the fast fraction and sub-minutes for the slow fraction (Vazquez et al., 2006; Matsuoka and Ueda, 2018; Banerjee et al., 2023). As discussed above, the membrane association is promoted by PI(4,5)P_2_ and the dissociation is promoted by PI(3,4,5)P_3_. Upon generation of the PI(3,4,5)P_3_-enriched domain through the activation of Ras and PI3K, PTEN located inside the domain dissociates from the membrane faster than outside the domain, increasing the concentration in the cytoplasm. This effect causes an increase in the membrane association rate of PTEN, which is faster outside the PI(3,4,5)P_3_-enriched domain than inside the domain. As a consequence, the simultaneous generation of multiple PI(3,4,5)P_3_-enriched domains is suppressed. Therefore, although the domains can arise from the bistable nature of PTEN and PI(3,4,5)P_3_, unity of the domain, and thus cellular polarity, may be affected by the affinity of the PI(3,4,5)P_3_-independent interactions between PTEN and the cell membrane, the copy number of PTEN expressed in the cell, and the volume of the cytoplasm, in addition to the dynamics of the Ras excitable system.

### 3.4 Correlation between excitability and cell motility

There is growing evidence supporting the excitability of Ras is quantitatively correlated to cellular motility. In cell populations, the fraction of cells exhibiting traveling waves correlates with the mean migration speed. In wild-type *D. discoideum* cells grown in axenic medium and starved for 3–4 h, the fraction of cells exhibiting traveling waves is about 80%, and the mean migration speed is about 10 μm/min (Arai et al., 2010). Treatment with LY294002, a PI3K inhibitor, reduces the fraction in a concentration-dependent manner, reducing the mean migration speed similarly (Arai et al., 2010). A cultivation of cells in the presence of fendiline, an acid sphingomyelinase inhibitor, also reduces the fraction and mean migration speed (Shin et al., 2023). Both the fraction and speed are recovered by the exogenous addition of sphingomyelinase, wash-out of the inhibitor, or both; with the recovery increasing in this order (Shin et al., 2023). LY294002 and fendiline are thought to suppress the excitability of Ras by blocking the positive feedback from PI(3,4,5)P_3_ to Ras-GTP and by reducing membrane-bound Ras-GTP, as described below, respectively. Thus, the fraction of cells showing traveling waves serves as an indicator of the excitability and predicts an average cell motility in an ensemble of cells.

At the single-cell level, a traveling wave in an individual cell is characterized temporally by a period and spatially by the domain size. The periods measured with RBD_Raf1_-GFP and PHD_PKB/Akt_-GFP in wild-type *D. discoideum* cells average about 3–4 min (Arai et al., 2010). This finding indicates the time interval between two successive excitations and seems roughly equivalent to the lifetime of directional persistency in cell migration. In spontaneously migrating cells, when an excitation takes place locally on the cell membrane, a pseudopod projection is initiated to drive directed cell migration for a while. When the next excitation takes place at the rest of the membrane, the pseudopod is taken over by the newly formed lateral pseudopod to change the moving direction. A statistical analysis of velocity autocorrelation in the migration trajectory made by tracking the centroid of the cell revealed a characteristic time of 3.8 min, suggesting that the cells tend to migrate in the same direction for this time duration (Takagi et al., 2008). The oscillatory behavior of the cellular morphology produced by the alternating protrusion of pseudopodia has been clarified (Abercrombie et al., 1970; Killich et al., 1993; Shenderov and Sheetz, 1997; Maeda et al., 2008), and it is now understood that the oscillator is in the Ras excitatory system.

The domain size is correlated to the spatial size of the leading edge in migrating cells. The Ras-GTP-enriched domain or PI(3,4,5)P_3_-enriched domain occupies about one-third (120^o^) of the circular periphery of the spherical cell observed at the equatorial plane under CLSM (Arai et al., 2010; Matsuoka and Ueda, 2018; Fukushima et al., 2019). By disrupting *ptenA*, a unique gene encoding PTEN in *D. discoideum*, the domain size reaches a maximum (360^o^), and PI(3,4,5)P_3_ accumulates throughout the cell membrane (Iijima and Devreotes, 2002). Pseudopodia extension is activated on the whole cell membrane without directional preference, and thus the cells are hardly displaced in any direction (Matsuoka and Ueda, 2018). When endogenous PTEN is substituted with *H. sapiens* PTEN in *D. discoideum* cells by *ptenA* disruption and *H. sapiens* PTEN overexpression, the size of the PI(3,4,5)P_3_-enriched domain increases to about 180^o^ without intervening the traveling wave propagation (Matsuoka and Ueda, 2018). An area of the leading edge is also enlarged, where multiple pseudopodia are simultaneously extended and disturb directed migration, though the anterior-posterior polarity is established. The intermediate phenotype is caused because *H. sapiens* PTEN localizes less than endogenous PTEN at the membrane (Vazquez et al., 2006; Yang et al., 2015). A similar correlation between the domain size and cellular morphology was confirmed by manipulating the membrane localization of Inp54p, which catalyzes the dephosphorylation of PI(4,5)P_2_ into PI(4)P, a GEF domain of Rap1 GEF (RasGEFU, also known as GbpD), or the membrane localization of a constitutively active Rap1 or RasC with a chemically inducible dimerization system (Miao et al., 2017). After the forced membrane localization, the cells stochastically exhibited transitions of their migration modes from amoeboid into keratocyte-like movement, which was marked by a highly directed migration driven by a broad pseudopod and fan-shaped morphology (Miao et al., 2017). Some cells also transitioned to the oscillatory mode, in which they repeatedly exhibited expansion and retraction of the adhesion area (Miao et al., 2017). In the excitable system of Ras, certain RasGEF or RasGAP should be responsible for these temporal and spatial regulations of the Ras-GTP-enriched domain, but which specific GEFs and GAPs are unknown.

## 4 Spatiotemporal regulation of the excitable system

### 4.1 Intrinsic factors affecting excitable dynamics and cell migration

Intrinsic cell-to-cell differences in excitable dynamics can arise due to variations in the concentrations of the components of the common molecular network of the excitable system. In an ensemble of cells that are genetically identical and grown in the same medium, three different dynamics are seen in individual cells (no domain, transient domain, and traveling wave) (Arai et al., 2010). The spatiotemporal properties of the traveling wave, such as the period and domain size, also vary cell to cell. A numerical simulation predicted which parameter is responsible for the variability, as described above (Arai et al., 2010; Shibata et al., 2012; Fukushima et al., 2019). The origin of the variable concentration most likely lies in the transcription, translation, post-translational modification, subcellular localization and proteolysis of each component of the network.

In addition to proteins, membrane lipids, such as sphingomyelin and phosphatidylserine, regulate the excitable system (Shin et al., 2023). A cultivation of cells for 1–2 days in the presence of fendiline causes an accumulation of sphingomyelin on the cell membrane (Shin et al., 2023). Many of these cells show the dynamics of excitation instead of oscillation even in the presence of caffeine (Shin et al., 2023). This observation suggests that the membrane sphingomyelin level negatively regulates the excitability of Ras. Eliminating the accumulated sphingomyelin by the exogenous addition of sphingomyelinase purified from *Staphylococcus aureus* recovers the traveling wave (Shin et al., 2023). The exogenous addition of phosphatidylserine also recovers the traveling wave (Shin et al., 2023). Electrostatic interactions between mammalian K-Ras and phosphatidylserine enhance the membrane localization and signaling activity of K-Ras [Zhou et al., 2021]. Similarly, phosphatidylserine may positively regulate the excitability by increasing the Ras-GTP density on the cell membrane in *D. discoideum* cells. It was proposed that the phosphatidylserine level on the cell membrane is regulated via the sphingomyelin level in an anti-correlative manner (Cho et al., 2015), but the mechanism is currently unknown. These results imply that membrane lipids, whose compositions likely depend on the metabolic state of the cell, serve as regulators of the Ras excitable system to connect the metabolic state of the cell to its behavior.

### 4.2 Extrinsic regulation for chemotaxis

The excitability of Ras is spatiotemporally modulated by extracellular cues, such as a chemoattractant gradient, so that the Ras-GTP-enriched domain is generated at the side facing the higher concentration for chemotaxis. cAMP, a chemoattractant of *D. discoideum*, is detected by 7-transmembrane G-protein-coupled receptors (Jin et al., 2008). Ligand binding leads to the activation of coupled trimeric G-protein, whose α and βγ subunits individually activate downstream parallel signaling pathways including RasG/D-PI3K, RasC-TorC2, Rap1/cGMP and PLA2 [Devreotes et al., 2016; Janetopoulos et al., 2001; Veltman et al., 2008]. The difference in the ligand concentration is detected using a local excitation global inhibition (LEGI) model, which is based on a molecular network and feedforward control (Iglesias and Devreotes, 2008; Parent and Devreotes, 1999; Takeda et al., 2012). The LEGI model assumes three kinds of molecules: an excitor, an inhibitor and a response regulator. Upon ligand binding to the receptor, the excitor and inhibitor are produced in equal amounts depending on the ligand concentration. However, the production rate of the excitor is faster than the inhibitor, and the balance between their amounts is processed and transmitted by the response regulator. Considering the response of *Dictyostelium* cells toward a spatially uniform increase in extracellular cAMP concentration, the amount of the response regulator becomes positive transiently and equals 0 after enough time has passed, where neither the excitor nor inhibitor exhibits an adaptation but the response regulator does. The peak value of the response regulator varies depending on the ligand concentration. Assuming that the response regulator activates Ras, when the amount of the response regulator crosses the threshold, Ras will exhibit excitation (Shibata et al., 2013; Bhattacharya and Iglesias, 2018). According to the characteristics of the excitable system, the spatial size of the Ras-GTP-enriched domain is expected to depend on the concentration of cAMP, and the response amplitude of Ras-GTP is constant (Shibata et al., 2013). Such characteristics were experimentally confirmed and showed the excitation on more than half of the cell membrane evoked an EC_50_ of 0.2–1 nM (Nishikawa et al., 2014; Shin et al., 2023). Signaling molecules under the regulation of Ras-GTP exhibit the response accordingly, including transient PI(3,4,5)P_3_ production, PI(4,5)P_2_ reduction, and PTEN detachment from the membrane (Fukushima et al., 2019; Devreotes et al., 2016). Importantly, in this model, the transient activation of the signaling molecules upon uniform cAMP stimulation ceases autonomously due not to an adaptation but to the nature of the excitable system.

To explain the localized response under the concentration gradient, the spatial properties of the excitor and inhibitor are taken into account: the excitor stays relatively longer on the cell membrane, while the inhibitor diffuses relatively quickly in the cytoplasm (Postma and Haastert, 2001; Levchenko and Iglesias, 2002). After production of the excitor and inhibitor is saturated, the excitor retains information on the ligand concentration at both sides facing the higher and lower concentrations of cAMP, while the inhibitor only retains information of the average concentration due to diffusion in the cytoplasm. The difference between the amount of the excitor and the inhibitor is positive and negative at the higher and lower concentration sides, respectively, and thus the response regulator is activated and inactivated at these sides. Thus, the excitability of Ras is enhanced only at the higher concentration side (Shibata et al., 2013). In fact, in cells treated with latrunculin A, the PI(3,4,5)P_3_-enriched domain and the Ras-GTP-enriched domain are generated continuously at the side facing the tip of a micropipette containing cAMP (Xu et al., 2006; Zhang et al., 2008; Wang et al., 2013; Kamimura et al., 2016). However, it should be noted that Ras-GTP and PI(3,4,5)P_3_ exhibit oscillatory dynamics in the excitable system. To generate a sustained response at the higher concentration side, some kind of bistable behavior is required. The excitable system generally exhibits bistable behavior depending on the condition, and the signaling molecules that activate Ras upon cAMP stimulation may switch the dynamics to a bistable one. Otherwise, the positive feedback on Ras-GTP from PI(3,4,5)P_3_ that exhibits bistability may be enhanced by cAMP. Although there are several reports showing RasGEF and RasGAP are involved in chemotactic signaling, such as RasGEFR and NF1 (Kae et al., 2004; Zhang et al., 2008), the identification of more Ras regulators is necessary to understand the molecular mechanism driving robust chemotactic responses against a wide range of cAMP concentrations (Kamimura et al., 2016; Miyanaga et al., 2018).

**FIGURE 5:**
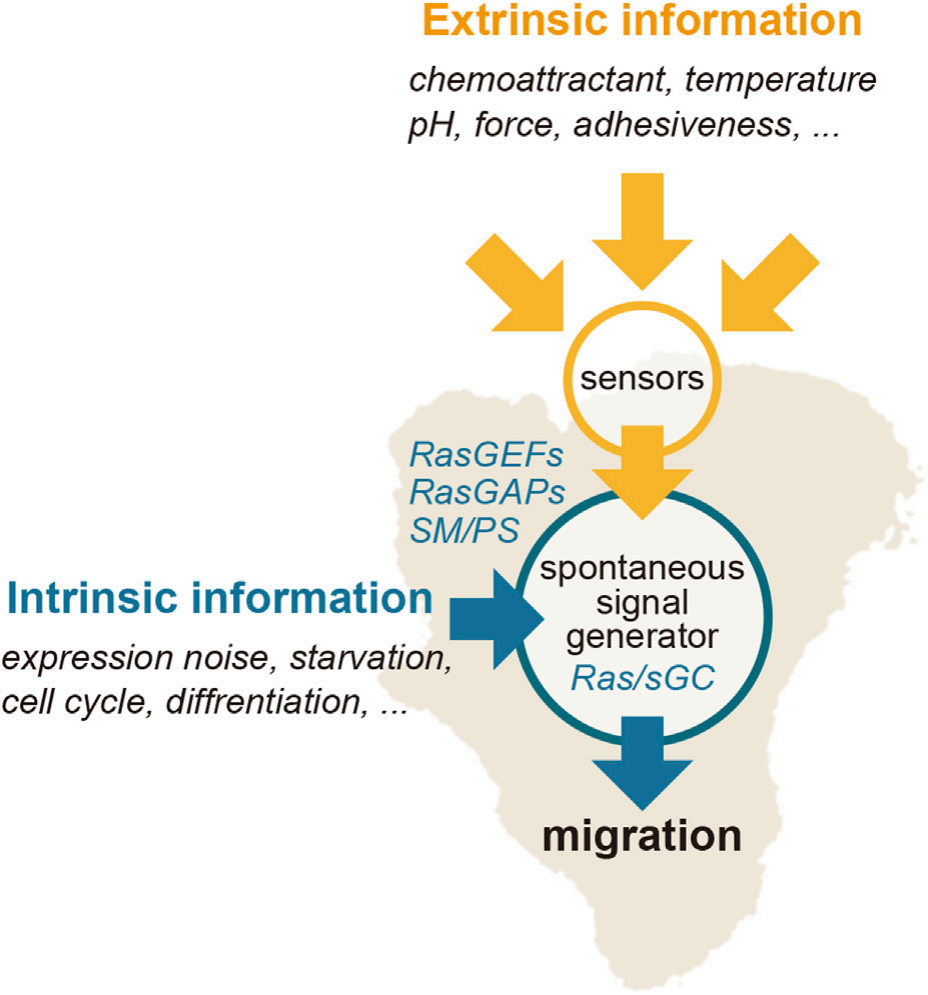
Regulation of the Ras excitable system. Extrinsic information, such as a chemoattractant gradient, can bias the spontaneously generated signal of the Ras-GTP-enriched domain spatiotemporally.

The current understanding of chemotactic signaling is based on mostly two systems; an excitable system that operates the spontaneous generation of the Ras-GTP-enriched domain, and a LEGI system that operates for sensing extracellular chemical gradients (Figure 3). The configuration of these systems is essentially the same as those previously proposed by Fumio Oosawa, who examined how cellular responses are generated from the combination of a sensor and spontaneous signal generator (Oosawa, 2001; Oosawa, 2007) (Figure 5). Cells sometimes exhibit deterministic or reflective responses upon certain kinds of stimulations, in which the same responses are observed every time in all cells. In this case, the effect of fluctuations in the spontaneous signal is relatively small, and the external stimulation detected by the sensor is directly coupled to the cell’s behavior. On the other hand, probabilistic responses are sometimes observed, in which the responses to the same stimulation vary over time or from cell to cell. In this case, the activity of the spontaneous signal generator is different depending on the cellular state. The essence of this framework lies in the activity of the spontaneous signal generator, which generates the signal without any external stimulation for the spontaneous behavior and can be regulated by environmental cues, if they exist, for environmental adaptation. Considering that the sensing systems for stimulations other than chemoattractants may operate similarly for cellular migratory behavior, the spontaneous signal generator may offer a platform for integrating extracellular information to make decisions in cell motility.

Excitable systems are noise-driven in that internal fluctuations of the components in the system can cause the excitation. In the case of chemotactic signaling systems, the internal noise-induced excitation leads to the spontaneous generation of the Ras-enriched domain on the membrane. Environmental noise may also affect the dynamics of the excitable system. In experimental observations of *Dictyostelium* chemotaxis, the cells can detect a faint signal under shallow gradients (Mato et al., 1975; Fisher et al., 1989; van Haastert 1995; Song et al., 2006; Ohtsuka et al., 2021). Stochastic fluctuations of the chemoattractant-receptor interactions inevitably cause noise in the chemotactic signals. A mathematical model predicted that the signal-to-noise ratio of the chemotactic signals at the chemoattractant receptors determines the efficiency of the chemotaxis (Ueda and Shibata, 2007; Amselem et al., 2012). This fluctuation-based model also shows that the threshold gradient for chemotaxis (ΔC_threshold_) is proportional to the square root of the mean concentration (C^0.5^) (Haastert, 1983; Ueda and Shibata, 2007; Haastert and Postma, 2007). How the signaling system operates reliably under stochastic fluctuations is a fundamental question in general. Also, the physiological significance of cellular decision-making being driven by noise-driven excitatory systems is not fully understood. The input-output relationship in the excitable system also remains to be clarified. Furthermore, cell motility exhibits randomness in the absence of guidance cues and even in environments where sharp gradients of chemoattractants and electric fields are applied (Takagi et al., 2008; Sato et al., 2009). Finally, randomness in cell motility may assist a cell in transversing complex environments, such as those with obstacles, by adjusting the spatiotemporal dynamics of the excitatory system (Nishimura et al., 2009; Nishimura et al., 2012).

### 4.3 Coordination between multiple excitable systems

Multiple excitable systems are involved in the generation of domains enriched with anterior signaling molecules and thus the pseudopod dynamics. Among the three signaling pathways working in parallel to RasG-PI3K, the Rap1/cGMP pathway exhibits excitable dynamics (Tanabe et al., 2018). The two excitable systems have similarities and differences. Similar to Ras-GTP and PI(3,4,5)P_3_, soluble guanylyl cyclase (sGC) localizes to the leading-edge membrane in migrating cells and to the whole cell membrane in response to uniform cAMP stimulation through the N-terminal domain (Veltman et al., 2005). The response to cAMP by sGC and PI(3,4,5)P_3_ is observed in cells lacking PI3K activity and the sGC N-terminal domain, respectively (Tanabe et al., 2018), suggesting that PI(3,4,5)P_3_-enriched and sGC-enriched domains arise independently of each other to activate pseudopod formation. Unlike RasG-PI3K excitation, F-actin is indispensable for sGC excitation (Tanabe et al., 2018). In addition, the length of the refractory period is different between the Rap1/cGMP excitable system (10–20 s) and the RasG-PI3K system (∼60 s) (Tanabe et al., 2018; Nishikawa et al., 2014). At the onset of the excitation, sGC localizes to the membrane through F-actin binding and produces cGMP (Tanabe et al., 2018). cGMP directly binds to and activates GbpC, which in turn destabilizes F-actin and suppresses pseudopod formation (Haastert et al., 2021; Bosgraaf et al., 2002). Thus, cGMP mediates the delayed negative feedback loop to bring the system back to the resting state, and its concentration is a major determinant of the length of the refractory period. Due to the faster cycling of the excitable dynamics, sGC activates the pseudopod projection more frequently than PI(3,4,5)P_3_, and thus sGC is detected at the leading edge more frequently than PI(3,4,5)P_3_ (Tanabe et al., 2018). Upon the excitation of sGC, the excitation of Ras and PI(3,4,5)P_3_ can be induced through F-actin via positive feedback against Ras (Sasaki et al., 2007), and pseudopods with both sGC and PI(3,4,5)P_3_ localization exhibit prolonged elongation compared to those with sGC or PI(3,4,5)P_3_ localization (Tanabe et al., 2018). Therefore, a combination of the two excitable systems ensures variations in the pseudopod dynamics to ensure flexible responses to complex environments.

Collective migration in multicellular structures is another migration mode observed in an evolutionally wide range of organisms (Friedl and Gilmour, 2009). *Dictyostelium discoideum* has long been a model organism for collective cell migration (Weijer, 2009; Goldbeter, 2017). The migration is governed by traveling waves of extracellular cAMP propagating within a cell mass (Bonner and Savage, 1947; Tomchik and Devreotes, 1981). When starved, tens of thousands of cells aggregate autonomously to form a multicellular structure (Raper, 1984). The cells located at the aggregation center secrete cAMP synchronously every 3–6 min (Alcantara and Monk, 1974; Gregor et al., 2010). cAMP stimulates nearby cells to exhibit chemotaxis toward the aggregation center and at the same time to produce and secrete cAMP themselves (Roos et al., 1977). The secreted cAMP induces the same responses in outwardly neighboring cells but not inwardly neighboring cells due to refractoriness (Gerisch et al., 1975). Thus, a high concentration of extracellular cAMP propagates as concentric or spiral traveling waves outwardly, and the cells exhibit chemotaxis toward the aggregation center inwardly (Tomchik and Devreotes, 1981; Hashimura et al., 2019). The cAMP wave is based on an excitable system that involves the same signaling molecules as those of the RasG-PI3K pathway, such as RasG, PI3K and PI(3,4,5)P_3_ (Martiel and Goldbeter, 1987; Maeda et al., 2004; Gregor et al., 2010); and these two excitable systems can be coupled to each other during collective migration. In fact, under normal conditions, PI(3,4,5)P_3_ exhibits excitation oscillations in synchronicity with the cAMP wave (Dormann and Weijer, 2006; Hashimura et al., 2019). In this process, talin B, a talin homolog connecting the cytoskeleton and extracellular space, suppresses PI3K to create random migration and thus collective migration (Yamazaki et al., 2020). Traveling waves are also observed in the intracellular signaling activity of ERK propagating across two-dimensionally arrayed Mardin-Darby canine kidney (MDCK) cells undergoing collective migration during wound healing (Aoki et al., 2017; Hino et al., 2020), suggesting a conserved mechanism of collective cell migration based on excitability. In summary, crosstalk and coupling among multiple excitable systems most likely underlie the various migration modes achieved in changing environments.

## 5 Conclusion

Excitable systems provide machinery for spontaneous signal generation in cell migration. They operate by utilizing fluctuations in the levels of the systems’ molecular components. This operating principle is the basis for cellular state-dependent responses to a stimulation, as some cells faithfully migrate toward the chemoattractant source, while others migrate randomly. Such spontaneous activity is generated via the spatiotemporal dynamics of the excitable system that emerge from the cellular state. It is likely that the same operating principle is the basis for the flexible responses cells demonstrate to complicated stimulations found in natural environments. In the presence of sometimes contradictory inputs, including chemical compounds, pH, shear stress, adhesiveness, heat and electric fields, excitable systems process multiple inputs from the environment to determine which direction the cell will move. For example, *D. discoideum* cells located at the middle of two aggregation centers sense two chemoattractant gradients whose directions are opposite to each other. Nevertheless, these cells do not stop their migration and move toward either of the aggregation centers. In this case, the aggregation center is likely to be probabilistically chosen based on which input signal crosses the threshold of the excitable system. Such uncertainty is acceptable from the viewpoint of cell survival; if the cells stop migration, they never pass their genome to their descendants. Regarding the driving force of the spontaneous signal generation, fluctuations in molecular levels, which inevitably accompany enzymatic reactions, such as phosphorylation and nucleotide exchange, are central. To understand the principles of the decision-making for cell motility, further investigation is awaited to identify the whole network structure that utilizes molecular fluctuations and integrates information.
